# Prediction of extended high viremia among newly HIV-1-infected persons in sub-Saharan Africa

**DOI:** 10.1371/journal.pone.0192785

**Published:** 2018-04-03

**Authors:** Kimberly A. Powers, Matthew A. Price, Etienne Karita, Anatoli Kamali, William Kilembe, Susan Allen, Eric Hunter, Linda-Gail Bekker, Shabir Lakhi, Mubiana Inambao, Omu Anzala, Mary H. Latka, Patricia E. Fast, Jill Gilmour, Eduard J. Sanders

**Affiliations:** 1 Department of Epidemiology, Gillings School of Global Public Health, The University of North Carolina at Chapel Hill, Chapel Hill, North Carolina, United States of America; 2 International AIDS Vaccine Initiative, New York, New York, United States of America; 3 Department of Epidemiology and Biostatistics, University of California San Francisco, San Francisco, California, United States of America; 4 Project San Francisco, Kigali, Rwanda; 5 Uganda Research Unit on AIDS, Uganda Virus Research Institute, Entebbe, Uganda; 6 Zambia-Emory Research Project, Lusaka & Copperbelt, Zambia; 7 Department of Pathology and Laboratory Medicine, Emory University, Atlanta, Georgia, United States of America; 8 Desmond Tutu HIV Centre, University of Cape Town, Cape Town, South Africa; 9 KAVI-ICR University of Nairobi, Nairobi, Kenya; 10 The Aurum Institute, Johannesburg, South Africa; 11 Imperial College of Science, Technology and Medicine, London, United Kingdom; 12 Kenya Medical Research Institute, Kilifi, Kenya; 13 University of Oxford, Headington, United Kingdom; 14 University of Amsterdam, Amsterdam, The Netherlands; University of Pittsburgh Centre for Vaccine Research, UNITED STATES

## Abstract

**Objective:**

Prompt identification of newly HIV-infected persons, particularly those who are most at risk of extended high viremia (EHV), allows important clinical and transmission prevention benefits. We sought to determine whether EHV could be predicted during early HIV infection (EHI) from clinical, demographic, and laboratory indicators in a large HIV-1 incidence study in Africa.

**Design:**

Adults acquiring HIV-1 infection were enrolled in an EHI study assessing acute retroviral syndrome (ARS) symptoms and viral dynamics.

**Methods:**

Estimated date of infection (EDI) was based on a positive plasma viral load or p24 antigen test prior to seroconversion, or the mid-point between negative and positive serological tests. EHV was defined as mean untreated viral load ≥5 log_10_ copies/ml 130–330 days post-EDI. We used logistic regression to develop risk score algorithms for predicting EHV based on sex, age, number of ARS symptoms, and CD4 and viral load at diagnosis.

**Results:**

Models based on the full set of five predictors had excellent performance both in the full population (c-statistic = 0.80) and when confined to persons with each of three HIV-1 subtypes (c-statistic = 0.80–0.83 within subtypes A, C, and D). Reduced models containing only 2–4 predictors performed similarly. In a risk score algorithm based on the final full-population model, predictor scores were one for male sex and enrollment CD4<350 cells/mm^3^, and two for having enrollment viral load >4.9 log_10_ copies/ml. With a risk score cut-point of two, this algorithm was 85% sensitive (95% CI: 76%-91%) and 61% specific (55%-68%) in predicting EHV.

**Conclusions:**

Simple risk score algorithms can reliably identify persons with EHI in sub-Saharan Africa who are likely to sustain high viral loads if treatment is delayed. These algorithms may be useful for prioritizing intensified efforts around care linkage and retention, treatment initiation, adherence support, and partner services to optimize clinical and prevention outcomes.

## Introduction

Antiretroviral therapy (ART) initiated early in HIV-1 infection preserves immune function [1], reduces adverse clinical outcomes [2–4], and prevents transmission [4]. Recognizing these benefits, HIV treatment guidelines recommend ART initiation at diagnosis, regardless of CD4 count [5,6]. However, as of July 2017, only 60% of low- and middle-income countries had adopted the “Treat All” policy, and only 9% had implemented this approach in a majority of treatment sites [7]. These lags in guideline implementation—combined with suboptimal care linkage among those who are ART-eligible at diagnosis [8, 9] and poor care retention both before [10] and after [11] ART initiation—result in substantial losses of both clinical and transmission prevention benefits.

Delayed ART initiation and poor retention are particularly detrimental among persons who sustain high viral loads in the absence of treatment, as they are at especially high risk of onward transmission [12] and disease progression [13,14]. If persons likely to sustain high viremia could be identified at HIV diagnosis, then intensified efforts to support care linkage, ART initiation, partner services, care retention, and ART adherence specifically among these persons could optimize clinical and transmission prevention benefits. We therefore sought to develop risk score algorithms for identifying newly HIV-infected cases likely to have extended high viremia (EHV)–that is, likely to sustain viral loads ≥5 log_10_ more than three months after HIV-1 acquisition—based on demographic, clinical, and laboratory indicators in a large cohort of HIV-1 seroconverters in Africa.

## Methods

### Study design, setting, population, and procedures

As described previously [15], adults at risk of HIV-1 infection in eastern and southern Africa were enrolled into a multi-center cohort study across nine research centers in Kenya (Nairobi, Kilifi), Uganda (Entebbe, Masaka), Rwanda (Kigali), Zambia (Lusaka, Copperbelt), and South Africa (Rustenburg, Cape Town). Study volunteers were tested for HIV-1 monthly or quarterly (depending on site), including p24 antigen testing to detect infection before seroconversion. Blood collected at antibody-negative visits was saved to enable retrospective HIV RNA testing for acute HIV infection if a subsequent sample was found antibody-positive. Volunteers with incident HIV-1 infection detected between March 2005 and December 2011 were invited to enroll in an early HIV infection (EHI) study. The study was approved by the Kenya Medical Research Institute Ethics and Review Board, the Kenyatta National Hospital Ethical Review Committee of the University of Nairobi, the University of Cape Town Health Science Research and Ethics Committee, the Rwanda National Ethics Committee, the Uganda Virus Research Institute Science and Ethics Committee, the Uganda National Council of Science and Technology, the University of Zambia Research Ethics Committee, the Emory University Institutional Review Board, and the Bio-Medical Research Ethics Committee at the University of KwaZulu Natal. All volunteers provided written informed consent.

At the time of EHI cohort enrollment, volunteers identified ≤90 days after their estimated date of infection (EDI) were asked whether they had experienced eleven symptoms of acute retroviral syndrome (ARS) in the three months before HIV-1 detection: fever, headache, night sweats, myalgia, fatigue, skin rash, oral ulcers, pharyngitis, lymphadenopathy, diarrhea, and anorexia [16]. As described previously [17], blood was drawn at enrollment for *pol* gene viral subtyping and CD4 and viral load quantification. The EDI was defined as 10 days before the first positive viral load test if antibody and p24 tests were negative at the time of the detectable viral load, 14 days before the first positive p24 test if no previous viral load or antibody tests were positive, or the midpoint between the last negative and first positive HIV-antibody test in the absence of any p24- or RNA-positive samples [17]. Following EHI study enrollment, viral load and CD4 quantification was performed monthly in the first three months after the EDI, quarterly until two years post-EDI, and semiannually thereafter.

### Statistical analyses

EHI study participants were included in the current analysis if their enrollment visit was ≤90 days post-EDI, ARS symptoms were assessed at enrollment, and at least one viral load measurement was available for EHV calculation. Similar to prior studies [18,19], we defined EHV as a mean pre-ART viral load ≥5 log_10_ copies/ml during the period 130–330 days post-EDI. Viral load measurements taken after ART initiation were censored. In descriptive analyses, we examined the distributions (overall and by subtype) of key demographic and clinical variables, including numbers of ARS symptoms and EHV prevalence.

We constructed logistic regression models with EHV as the outcome, first without and then with stratification by HIV-1 subtype. The overall (subtype-nonspecific) analyses were meant to represent model use in locations in which there is not a single predominant subtype, whereas the subtype-specific analyses were intended for locations in which a particular subtype is known to be most prevalent. Subtype-specific analyses were limited to subtypes A, C, and D (excluding a small number of recombinants), the only subtypes for which we had sufficient numbers of volunteers. Based on previous studies of viremia and/or clinical progression [20–27], we included sex, age (≥30 vs. <30), and a dichotomized measure of the number of ARS symptoms (≥2 vs. <2) as predictor variables in each full model. We also included enrollment viral load (dichotomized at the median: >4.9 vs. ≤4.9 log_10_ copies/ml) and CD4 stratum (< 350 vs. ≥ 350 cells/mm^3^) as predictors. We conducted backward elimination with a stopping rule of p = 0.2 for each model. To assess the performance of the full and final models, we examined the c-statistic, representing the area under each model’s receiver operating characteristic (ROC) curve. C-statistics of <0.69 were considered poor, 0.7–0.79 acceptable, 0.8–0.89 excellent, and ≥0.9 outstanding [28].

After constructing predictive models overall and by HIV-1 subtype, we sought to develop a risk score algorithm for predicting EHV in each context. For each of the four final models (one for the overall population and one for each of the three HIV-1 subtypes), we calculated a predictor score for each explanatory variable by rounding its beta coefficient (i.e., natural log of the odds ratio) to the nearest integer. After calculating a risk score for each volunteer by summing the applicable predictor scores from a given model, we calculated the sensitivity and specificity of each possible risk score cut-point within each model. All analyses were conducted with SAS 9.4 (SAS Institute, Cary, NC).

## Results

**Fig 1:**
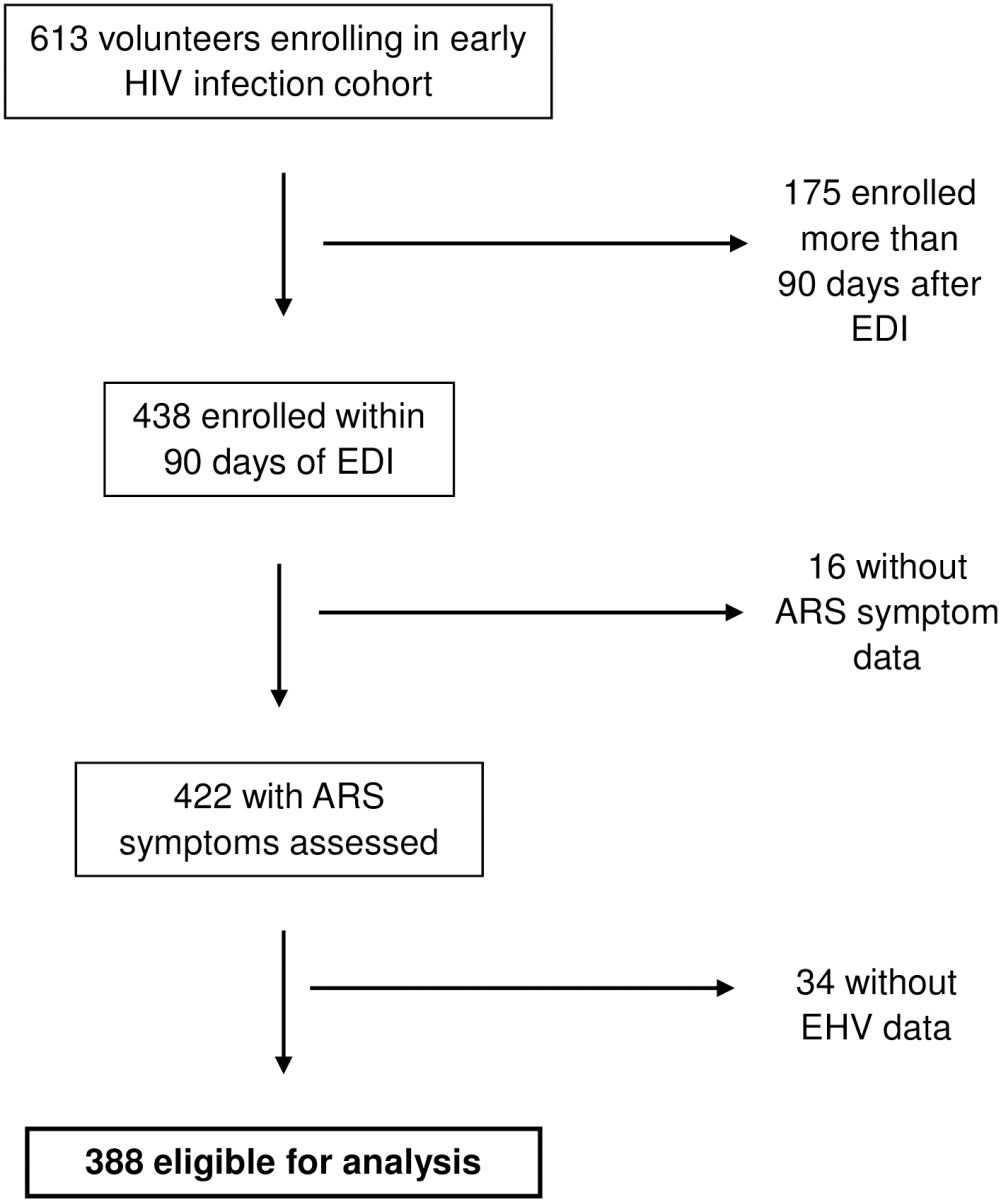
Study flow chart. EDI = estimated date of infection; ARS = acute retroviral syndrome; EHV = extended high viremia.

Of 613 HIV-1 seroconverters enrolling in the EHI cohort, 422 (68.8%) enrolled and completed information on ARS symptoms ≤90 days after their EDI (Fig 1). Of these, 388 (91.9%) had at least one pre-ART viral load measurement (range = 1–5 measurements; mode = 2 measurements) between 130 and 330 days post-EDI, and were thus eligible for analysis. Approximately one-third (34.8%) of the eligible volunteers had HIV-1 subtype A, 43.8% subtype C, and 15.7% subtype D, with the remaining 5.7% comprising other subtypes (Table 1). Across subtypes, the median age among males and females was 30 and 28 years, respectively, and the majority (72.9%) of volunteers belonged to serodiscordant couples. A lower proportion of those with subtype A were female versus those with subtypes C and D, and although the age distribution in females was similar across subtypes, male volunteers with subtype A were appreciably younger than those with subtype C (but not D). Due to differences in at-risk source populations across research centers, serodiscordant couples were more prevalent among those with subtype C than with the other two subtypes, and men who have sex with men were more prevalent among those with subtype A. Overall, the median time between EDI and enrollment was 45 days, median number of ARS symptoms was 2, and median enrollment viral load was 4.9 log_10_ copies/ml. Time since EDI and enrollment viral load were similar across subtypes, but subtype A volunteers had a greater number of ARS symptoms than those with subtypes C and D (as previously reported in [16]). The proportions with CD4>350 and CD4>500 (83.1% and 54.3% overall, respectively) were similar across subtypes.

**Table 1:** Characteristics at enrollment*.

Characteristic	Overall (N = 388)	Clade A (N = 135)	Clade C (N = 170)	Clade D (N = 61)	Other** (N = 22)
N (%) female^†^ ^‡^	152 (39.2)	38 (28.1)	78 (45.9)	29 (47.5)	7 (31.8)
Median (range) age, males^†^ ^¶^	30 (18–58)	28 (18–52)	33 (19–52)	30 (19–58)	30 (21–47)
Median (range) age, females	28 (16–53)	27 (19–53)	29 (16–45)	27 (17–46)	28 (18–42)
Number (%) in risk group: ^†^ ^‡^ ^¶^					
Serodiscordant couples	283 (72.9)	71 (52.6)	151 (88.8)	45 (73.8)	16 (72.7)
Men reporting sex with men	68 (17.5)	48 (35.6)	8 (4.7)	6 (9.8)	6 (27.3)
Other / don’t know	37 (9.5)	16 (11.9)	11 (6.5)	10 (16.4)	0 (0.0)
Median (IQR) days since EDI	45 (25–56)	43 (22–56)	45 (26–56)	50 (34–56)	43 (18–59)
Median (range) number of ARS symptoms^†^ ^‡^	2 (0–10)	4 (0–10)	1 (0–10)	1 (0–8)	2 (0–8)
Median (range) log_10_ viral load	4.9 (1.4–7.3)	4.9 (2.3–7.3)	4.9 (1.7–6.5)	4.9 (1.4–6.7)	4.9 (1.4–6.8)
N (% of those with CD4 data) with CD4>350	291 (83.1)	99 (79.2)	123 (85.4)	50 (83.3)	19 (90.5)
N (% of those with CD4 data) with CD4>500	190 (54.3)	74 (59.2)	71 (49.3)	36 (60.0)	9 (42.9)

* all within 90 days of estimated infection acquisition date.

** Includes 1 subtype B infection, 2 subtype G, 17 recombinant, and 2 with missing subtype.

^†^ Subtype A vs subtype C comparison statistically significant at α = 0.05.

^‡^ Subtype A vs subtype D comparison statistically significant at α = 0.05.

^¶^ Subtype C vs subtype D comparison statistically significant at α = 0.05.

**Fig 2:**
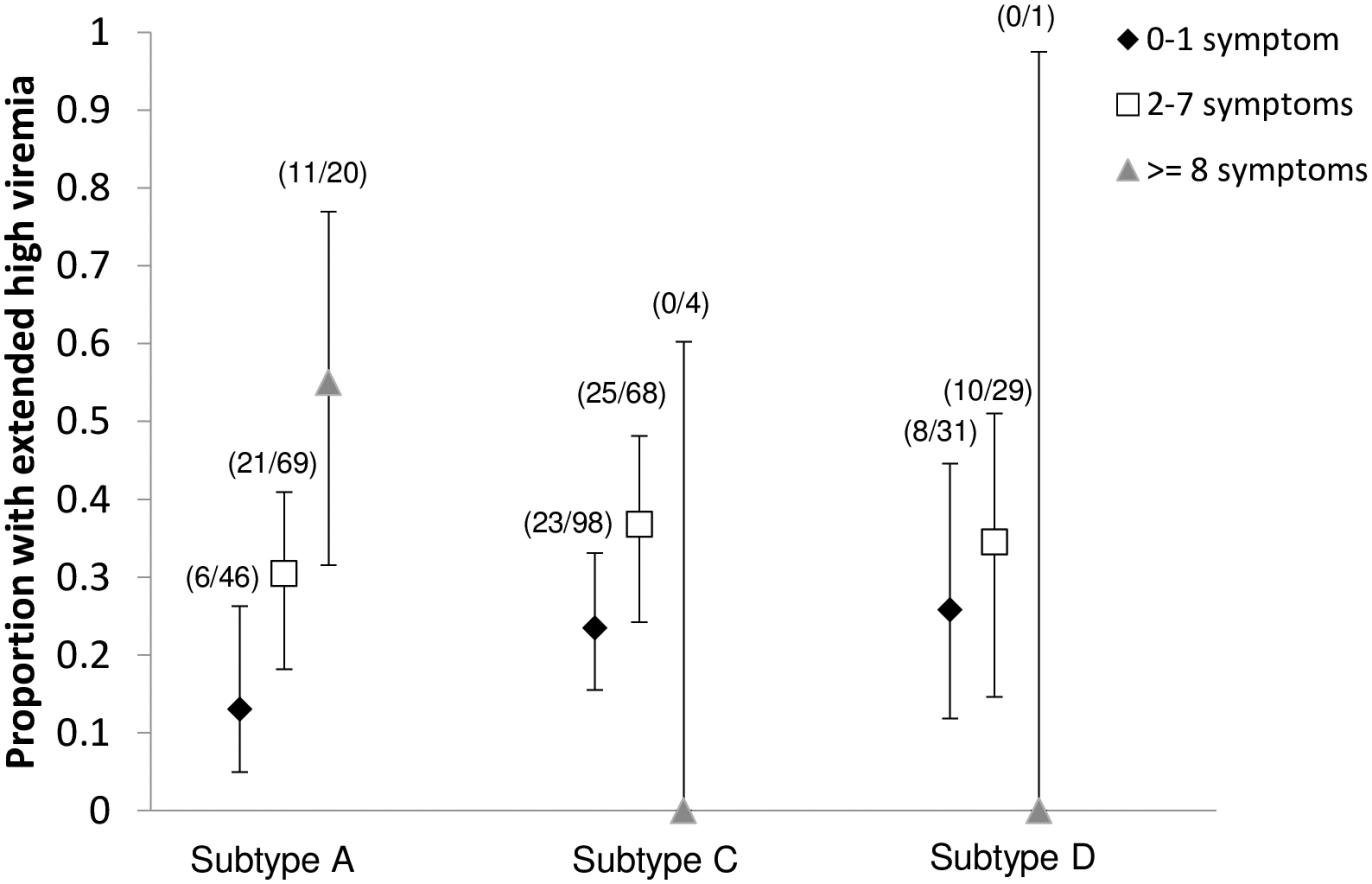
Extended high viremia prevalence by subtype and number of ARS symptoms. The points represent EHV prevalence for a given range of the number of symptoms; the brackets represent the 95% confidence intervals. The numerator and denominator for each proportion are shown in parentheses above each estimate.

The relationship between number of ARS symptoms and EHV prevalence varied across subtypes (Fig 2). Subtype A volunteers exhibited a clear increase in EHV prevalence as the number of ARS symptoms increased from 0–1 to 2–7 to ≥8. Subtype C and D volunteers also experienced an increase in EHV prevalence as the number of ARS symptoms increased from 0–1 to 2–7, but subtype D estimates were imprecise. No subtype C or D volunteers with ≥8 ARS symptoms experienced EHV, but very few subtype C and D volunteers had this many symptoms (N = 4 and N = 1, respectively).

Logistic regression models including sex, age, number of ARS symptoms, enrollment viral load, and enrollment CD4 had excellent performance both in the full population (c-statistic = 0.80) and when confined to persons with each of the three HIV-1 subtypes (c-statistic = 0.83, 0.83, and 0.80 for subtypes A, C, and D, respectively). The final models obtained through backward selection performed similarly (c-statistics of 0.79, 0.82, 0.83, and 0.80 in the full, subtype A, subtype C, and subtype D populations, respectively). The final model for the full population included sex, age at infection, enrollment viral load, and enrollment CD4; the final model for subtype A infection included number of ARS symptoms, age at infection, and enrollment viral load; the final model for subtype C infection included male sex, age at infection, enrollment viral load, and enrollment CD4; and the final model for subtype D included age at infection and enrollment viral load.

Using the beta coefficients from the final model applied in the full population, we developed a risk score algorithm in which male sex and enrollment CD4 <350 cells/mm^3^ were each assigned one point, and enrollment log_10_ viral load >4.9 log_10_ copies/ml was assigned two points (Table 2). Based on the beta coefficients in the model developed specifically for subtype A infection, we assigned one point to number of ARS symptoms ≥2 and to age ≥30, and three points to enrollment viral load >4.9 log_10_ copies/ml. Point values for the subtype C algorithm were one point for male sex and age ≥30, two points for enrollment viral load >4.9 log_10_ copies/ml, and two points for enrollment CD4 <350 cells/mm^3^. In the algorithm for subtype D, both enrollment viral load >4.9 log_10_ copies/ml and age <30 at infection carried two points on the basis of their beta coefficients.

**Table 2:** Model coefficients and corresponding predictor scores by HIV-1 subtype.

Model predictors:	All		Subtype A		Subtype C		Subtype D	
	β	points	β	points	β	points	β	points
≥2 ARS symptoms	--	--	1.01	1	--	--	--	--
Male sex	0.64	1	--	--	1.38	1	--	--
Age ≥ 30 at EDI	0.44	0	0.63	1	0.83	1	-1.67	-2*
Enrollment viral load > 4.9 log_10_ copies/ml	2.09	2	2.77	3	1.79	2	2.18	2
Enrollment CD4 < 350	0.60	1	--	--	2.00	2	--	--

-- Predictor not included in final model because p≥0.2

* To confine predictor scores to positive values in the subtype D model, we converted the point value of -2 for age ≥ 30 to a value of +2 for age < 30.

**Fig 3:**
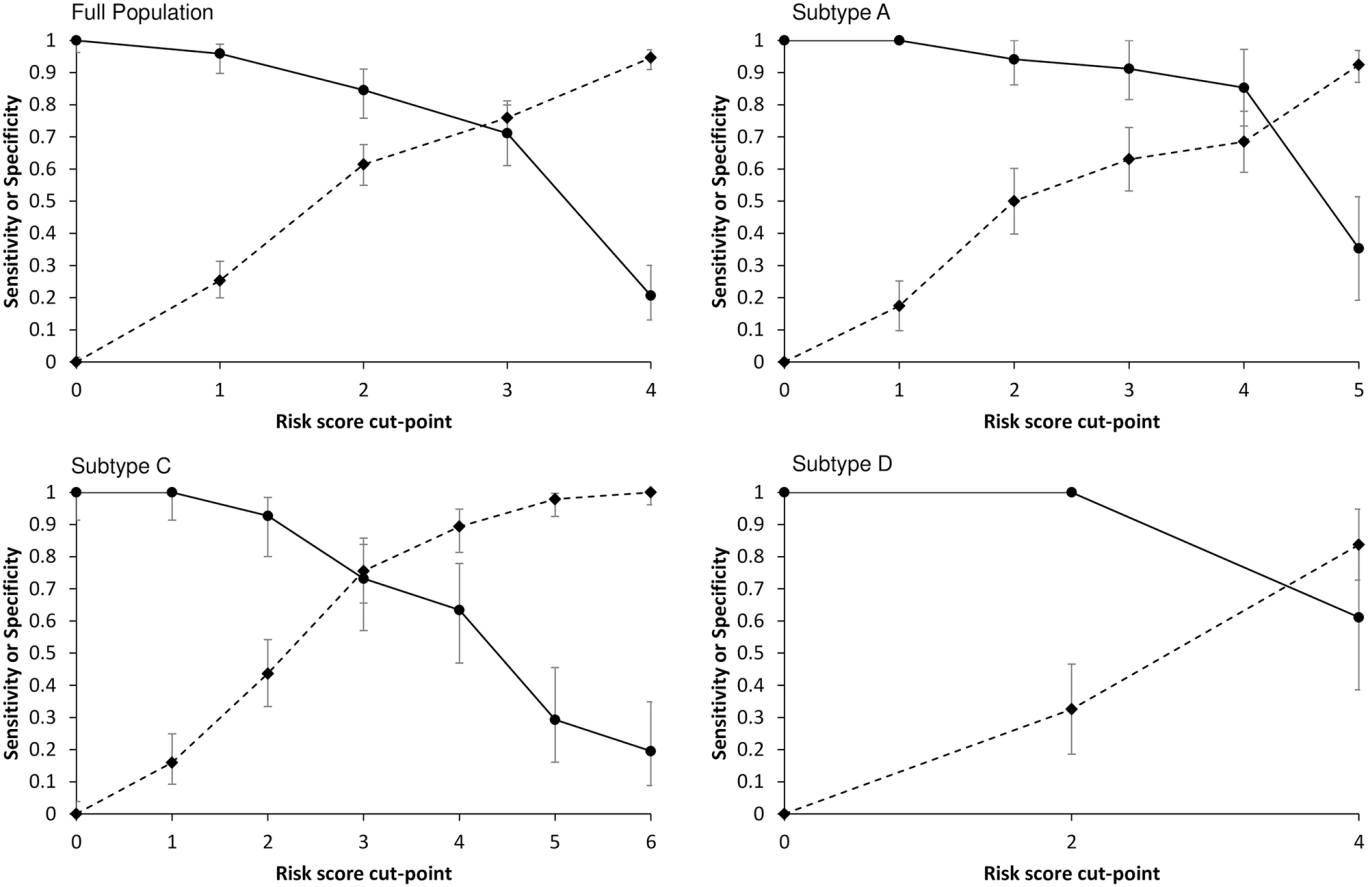
Sensitivity and specificity of risk score models developed in the full and subtype-specific populations. The horizontal axes display all possible risk score cut-points that could be chosen for clinical implementation of a given algorithm. In clinical implementation, all persons with risk scores at or above a chosen cut-point would be identified as likely to subsequently have extended high viremia. Circles represent the proportion of all EHV cases with scores at or above a given risk score cut-point (i.e., sensitivity). Diamonds represent the proportion of all those who did not have EHV with scores below a given risk score cut-point (i.e., specificity).

The overall and subtype-specific algorithms performed well (Fig 3). For example, with a cut-point of two in the full-population model, the sensitivity was 85% (95% CI: 76%-91%)–that is, the algorithm would correctly identify 85% of persons who would subsequently experience EHV. The corresponding specificity was 61% (55%-68%)–that is, 61% of those who did not ultimately experience EHV had scores <2. In other words, the algorithm would correctly rule out 61% of persons who would not experience EHV. In the subtype A model, a cut-point of three had a sensitivity of 91% (82%-100%) and a specificity of 63% (53%-73%); in the subtype C model, a cut-point of two had a sensitivity of 93% (80%-98%) and a specificity of 44% (33%-54%); and in the subtype D model, a cut-point of two had a sensitivity of 100% and a specificity of 33% (19%-47%).

## Discussion

Rapid identification and treatment of newly HIV-infected persons can have important clinical and public health benefits, but many persons with EHI are likely to have CD4 counts above current ART initiation thresholds in many sub-Saharan African countries [6,7], and suboptimal linkage and retention are prevalent even among treatment-eligible persons [8–11]. The detrimental effects of delaying treatment are particularly great among newly infected persons who sustain high viral loads, allowing unmitigated transmission for months or years before treatment begins. To determine whether limited resources for linkage, retention, treatment, and partner services could be efficiently targeted toward EHI cases with the highest potential for onward transmission and clinical progression, we sought to develop predictive models and risk score algorithms for EHV based on previously identified correlates of sustained high viremia.

The full predictive model containing number of ARS symptoms, male sex, age at infection, and enrollment CD4 and viral load had excellent performance in the overall and subtype-specific populations. This strong predictive ability was largely maintained in each of the more parsimonious, reduced models, suggesting that simple models with only 2–4 predictors could reliably identify candidates for intensified efforts around ART initiation, counseling, and monitoring in many sub-Saharan African settings.

Possible approaches for identifying potential EHI cases for algorithm application will vary across settings according to laboratory capabilities. Newly HIV-positive persons with recent HIV-negative test results (i.e., in the prior 3–6 months) should be considered as probable EHI cases, as should those with discordant rapid antibody tests in dual-test settings [29,30]. Though less commonly available, fourth-generation antibody/antigen tests or HIV RNA testing of antibody-negative persons offer more direct identification of EHI cases for EHV algorithm application.

The risk score algorithms that we developed both in the overall and subtype-specific populations performed well, suggesting that these algorithms could be useful in many sub-Saharan African diagnosis settings. In real-world implementation, clinical staff would complete a brief checklist of algorithm predictors for suspected or confirmed EHI cases and then sum predictor scores to calculate a patient’s risk score. For example, consider a forty-year-old male who tests HIV-antibody-positive after testing negative two months earlier in a setting where subtype A predominates. If this probable EHI case has four ARS symptoms and log_10_ viral load > 4.9 copies/ml, then he would have a risk score of five (sum of predictor scores = 1 for age + 1 for number of symptoms + 3 for viral load). Patients with a risk score above a chosen cut-point would then be selected for intensified efforts around care engagement, treatment initiation, and/or partner services.

Strengths of our study include the collection of relevant data under a standardized protocol and questionnaire at nine different sites across sub-Saharan Africa, as well as the relatively large population of newly HIV-infected persons arising from the multi-site design. We note, however, that the small numbers of volunteers within each subtype limited our ability to include a large number of predictors (e.g., each specific ARS symptom) in subtype-specific models. It is possible that subtype-specific algorithms based on particular symptoms or other variables would be more predictive. We also note that the timing of ARS assessment and viral load measurements within the course of HIV infection could differ in the real world, as detection of incident HIV occurred through regular testing in our population but may be more symptom-driven outside of the research context. It is difficult to predict the effect of such timing differences on symptom recall or viral load values, but any such effects could affect algorithm performance.

To our knowledge, only one other risk score algorithm relating ARS symptoms to longer-term viral load endpoints has been published [31]. That algorithm, which relied on expert opinion to select predictors and assign points (i.e., without a predictive model), included severe neurological symptoms (3 points), inpatient treatment (3 points), age ≥50 years (1 point), reported or documented fever (1 point), elevated liver enzymes (1 point), and thrombocytopenia (1 point). In applying the algorithm to persons infected predominantly with HIV-1 subtype B in Switzerland, higher risk scores were found to be associated with a higher set-point viral load after 90 days of untreated infection, but algorithm performance in terms of sensitivity, specificity, and c-statistic was not reported in the original study [31] or in a recent validation study in a US population [32]. Although the general purpose of the Swiss algorithm is similar to ours, the risk scores and results are not directly comparable, due to differences in the specific predictors included, participants’ HIV-1 subtype profiles, and study settings. In particular, the US and European contexts to which the previous algorithm is most likely to be generalizable is not subject to the same resource constraints as the settings in which our algorithm is intended to guide resource allocation.

Overall, our findings suggest that algorithms based on age, sex, CD4, viral load, and numbers of ARS symptoms could be useful in identifying newly HIV-infected persons in whom intensified efforts around ART initiation, retention, and/or partner services should be considered. By guiding efficient intervention targeting in resource-limited settings, such algorithms could enable important clinical and transmission prevention benefits from the earliest possible point in infection.

## References

[1] Le T, Wright EJ, Smith DM, He W, Catano G, Okulicz JF, et al Enhanced CD4+ T-cell recovery with earlier HIV-1 antiretroviral therapy. N Engl J Med. 2013; 368: 218–230. doi: 10.1056/NEJMoa1110187 23323898 10.1056/NEJMoa1110187 PMC3657555

[2] TEMPRANO ANRS 12136 Study Group, Daniel C, Moh R, Gabillard D, Badje A, Le Carrou J, et al A trial of early antiretrovirals and isoniazid preventive therapy in Africa. N Engl J Med. 2015; 373: 808–822. doi: 10.1056/NEJMoa1507198 26193126 10.1056/NEJMoa1507198

[3] INSIGHT START Study Group, Lundgren JD, Babiker AG, Gordin F, Emery S, Grund B, et al Intiation of antiretroviral therapy in early asymptomatic HIV infection. N Engl J Med. 2015; 373: 795–807. doi: 10.1056/NEJMoa1506816 26192873 10.1056/NEJMoa1506816 PMC4569751

[4] Cohen MS, Chen YQ, McCauley M, Gamble T, Hosseinipour MC, Kumarasamy N, et al Prevention of HIV-1 infection with early antiretroviral therapy. N Engl J Med. 2011; 365: 493–505. doi: 10.1056/NEJMoa1105243 21767103 10.1056/NEJMoa1105243 PMC3200068

[5] Thompson MA, Aberg JA, Cahn P, Montaner JS, Rizzardini G, Telenti A, et al Antiretroviral treatment of adult HIV infection: 2010 recommendations of the International AIDS Society—USA panel. JAMA. 2010; 304: 321–333. doi: 10.1001/jama.2010.1004 20639566 10.1001/jama.2010.1004

[6] WHO. Guideline on when to start antiretroviral therapy and pre-exposure prophylaxis for HIV. Geneva: World Health Organization, 2015 http://apps.who.int/iris/bitstream/10665/186275/1/9789241509565_eng.pdf 26598776

[7] WHO. Treat all: policy adoption and implementation status in countries. Geneva: World Health Organization, 2017 http://apps.who.int/iris/bitstream/10665/258538/1/WHO-HIV-2017.35-eng.pdf.

[8] Clouse K, Pettifor AE, Maskew M, Bassett J, Van Rie A, Behets F, et al Patient retention from HIV diagnosis through one year on antiretroviral therapy at a primary healthcare clinic in Johannesburg, South Africa. J Acquir Immune Defic Syndr. 2013; 62: e39–346. doi: 10.1097/QAI.0b013e318273ac48 23011400 10.1097/QAI.0b013e318273ac48 PMC3548953

[9] Plazy M, Dray-Spira R, Orne-Gleimann J, Dabis F, Newell ML. Continuum in HIV care from entry to ART initiation in rural KwaZulu-Natal, South Africa. Trop Med Int Health. 2014; 19:680–689. doi: 10.1111/tmi.12301 24654990 10.1111/tmi.12301

[10] Mugglin C, Estill J, Wandeler G, Bender N, Egger M, Gsponer T, et al Loss to programme between HIV diagnosis and initiation of antiretroviral therapy in sub-Saharan Africa: systematic review and meta-analysis. Trop Med Int Health. 2012; 17: 1509–20. doi: 10.1111/j.1365-3156.2012.03089.x 22994151 10.1111/j.1365-3156.2012.03089.x PMC3895621

[11] Fox MP, Rosen S. Retention of adult patients on antiretroviral therapy in low- and middle-income countries: systematic review and meta-analysis 2008–2013. J Acquir Immune Defic Syndr. 2015; 69: 98–108. doi: 10.1097/QAI.0000000000000553 25942461 10.1097/QAI.0000000000000553 PMC4422218

[12] Quinn TC, Wawer MJ, Sewankambo N, Serwadda D, Li C, Wabwire-Mangen F, et al Viral load and heterosexual transmission of human immunodeficiency virus type 1. Rakai Project Study Group. N Engl J Med. 2000; 342: 921–929. doi: 10.1056/NEJM200003303421303 10738050 10.1056/NEJM200003303421303

[13] Pedersen C, Katzenstein T, Nielsen C, Lundgren JD, Gerstoft J. Prognostic value of serum HIV-RNA levels at virologic steady state after seroconversion: relation to CD4 cell count and clinical course of primary infection. J Acquir Immune Defic Syndr Hum Retrovirol. 1997; 16: 93–99. 9358103 10.1097/00042560-199710010-00004

[14] Schacker T, Hughes J, Shea T, Coombs RW, Corey L. Biological and virologic characteristics of primary HIV infection. Ann Intern Med. 1998; 128: 613–620. 9537934 10.7326/0003-4819-128-8-199804150-00001

[15] Kamali A, Price MA, Lakhi S, Karita E, Inambao M, Sanders EJ, et al Creating an African HIV clinical research and prevention trials network: HIV prevalence, incidence, and transmission. PLoS One. 2015; 10: e0116100 doi: 10.1371/journal.pone.0116100 25602351 10.1371/journal.pone.0116100 PMC4300215

[16] Sanders EJ, Price MA, Karita E, Kamali A, Kilembe W, Bekker L-G, et al Differences in acute retroviral syndrome by HIV-1 subtype in a multicentre cohort study in Africa. AIDS. 2017; 31: 2541–2546. doi: 10.1097/QAD.0000000000001659 29028659 10.1097/QAD.0000000000001659 PMC5690309

[17] Amornkul PN, Karita E, Kamali A, Rida WN, Sanders EJ, Lakhi S, et al Disease progression by infecting HIV-1 subtype in a seroconverter cohort in sub-Saharan Africa. AIDS. 2013; 27: 2775–2786. doi: 10.1097/QAD.0000000000000012 24113395 10.1097/QAD.0000000000000012 PMC3815107

[18] Novitsky V, Ndung’u T, Wang R, Bussmann H, Chonco F, Makhema J, et al Extended high viremics: a substantial fraction of individuals maintain high plasma viral RNA levels after acute HIV-1 subtype C infection. AIDS. 2011; 25: 1515–1522. doi: 10.1097/QAD.0b013e3283471eb2 21505307 10.1097/QAD.0b013e3283471eb2 PMC3544358

[19] Campbell MS, Kahle EM, Celum C, Lingappa JR, Kapiga S, Mujugira A, et al Plasma viral loads during early HIV-1 infection are similar in subtype C- and non-subtype-C-infected African seroconverters. J Infect Dis. 2013; 207: 1166–70. doi: 10.1093/infdis/jit015 23315322 10.1093/infdis/jit015 PMC3583276

[20] Kelley CF, Barbour JD, Hecht FM. The relation between symptoms, viral load, and viral load set point in primary HIV infection. J Acquir Immune Defic Syndr. 2007; 45: 445–448. doi: 10.1097/QAI.0b013e318074ef6e 17514014 10.1097/QAI.0b013e318074ef6e

[21] Sullivan PS, Fideli U, Wall KM, Chomba E, Vwalika C, Kilembe W, et al Prevalence of seroconversion symptoms and relationship to set point viral load: findings from a subtype C epidemic, 1995–2009. AIDS. 2012; 26: 175–184. doi: 10.1097/QAD.0b013e32834ed8c8 22089380 10.1097/QAD.0b013e32834ed8c8 PMC3589587

[22] Lindback S, Karlsson AC, Mittler J, Blaxhult A, Carlsson M, Briheim G, et al Viral dynamics in primary HIV-1 infection. Karolinska Institutet Primary HIV Infection Study Group. AIDS. 2000; 14: 2283–2291. 11089616 10.1097/00002030-200010200-00009

[23] Kaufmann GR, Cunningham P, Zaunders J, Law M, Vizzard J, Carr A, et al Impact of early HIV-1 RNA and T-lymphocyte dynamics during primary HIV-1 infection on the subsequent course of HIV-1 RNA levels and CD4+ T-lymphocyte counts in the first year of HIV-1 infection. Sydney Primary HIV Infection Study Group. J Acquir Immune Defic Syndr. 1999; 22: 437–444. 10961604 10.1097/00126334-199912150-00003

[24] Donnelly CA, Bartley LM, Ghani AC, Le Fevre AM, Kwong GP, Cowling BJ, et al Gender difference in HIV-1 RNA viral loads. HIV Med. 2005; 6: 170–178. doi: 10.1111/j.1468-1293.2005.00285.x 15876283 10.1111/j.1468-1293.2005.00285.x

[25] Farzadegan H, Hoover DR, Astemborski J, Lyles CM, Margolick JB, Markham RB, et al Sex differences in HIV-1 viral load and progression to AIDS. Lancet. 1998; 352: 1510–1514. doi: 10.1016/S0140-6736(98)02372-1 9820299 10.1016/S0140-6736(98)02372-1

[26] Pezzotti P, Phillips AN, Dorrucci M, Lepri AC, Galai N, Vlahov D, et al Category of exposure to HIV and age in the progression to AIDS: longitudinal study of 1199 people with known dates of seroconversion. BMJ. 1996; 313: 583–586. 8806246 10.1136/bmj.313.7057.583 PMC2352019

[27] Alioum A, Leroy V, Commenges D, Dabis F, Salamon R. Effect of gender, age, transmission category, and antiretroviral therapy on the progression of human immunodeficiency virus infection using multistate Markov models. Epidemiology. 1998; 9: 605–612. 9799168

[28] Hosmer D. W. and Lemeshow S. Assessing the fit of the model In: Applied logistic regression, Second Edition Hoboken: John Wiley & Sons, Inc., 2000.

[29] Powers KA, Miller WC, Pilcher CD, Mapanje C, Martinson FE, Fiscus SA, et al Improved detection of acute HIV-1 infection in sub-Saharan Africa: development of a risk score algorithm. AIDS. 2007; 21: 2237–2242. doi: 10.1097/QAD.0b013e3282f08b4d 18090052 10.1097/QAD.0b013e3282f08b4d PMC2673577

[30] Wahome E, Fegan G, Okuku HS, Mugo P, Price MA, Mwashigadi G, et al Evaluation of an empiric risk screening score to identify acute and early HIV-1 infection among MSM in Coastal Kenya. AIDS. 2013; 27: 2163–2166. doi: 10.1097/QAD.0b013e3283629095 23842136 10.1097/QAD.0b013e3283629095 PMC3748854

[31] Braun DL, Kouyos R, Oberle C, Grube C, Joos B, Fellay J, et al A novel acute retroviral syndrome severity score predicts the key surrogate markers for HIV-1 disease progression. PLoS One. 2014; 9: e114111 doi: 10.1371/journal.pone.0114111 25490090 10.1371/journal.pone.0114111 PMC4260784

[32] Hoenigl M, Braun DL, Kouyos R, Gunthard HF, Little SJ. Evaluation of the predictive potential of the short acute retroviral syndrome severity score for HIV-1 disease progression in individuals with acute HIV infection. J Acquir Immune Defic Syndr. 2017; 74: e114–e116. doi: 10.1097/QAI.0000000000001263 28225720 10.1097/QAI.0000000000001263 PMC5324781

